# Selective Liquid–Liquid Extraction of Thorium(IV) from Rare-Earth Element Mixtures

**DOI:** 10.1021/acs.inorgchem.4c01240

**Published:** 2024-07-17

**Authors:** Wyatt B. Larrinaga, Bailey J. Lake, Victoria D. Pacanowski, Michael G. Patterson, Michael Hudson, Faith M. Carlson, Gabriel Heselschwerdt, Steven Balboa, Shannon M. Biros, Eric J. Werner

**Affiliations:** † Department of Chemistry and Biochemistry, The University of Tampa, 401 West Kennedy Boulevard, Tampa, Florida 33606, United States; ‡ Department of Chemistry, Grand Valley State University, 1 Campus Drive, Allendale, Michigan 49401, United States

## Abstract

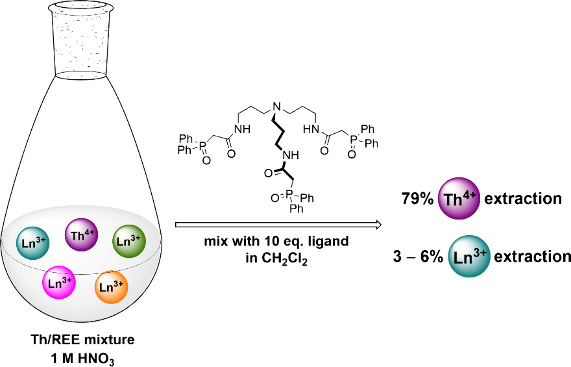

One of the major challenges in processing rare-earth element (REE) materials arises from the large amounts of radioactive thorium (Th) that are often found within REE minerals, encouraging enhanced metal separation procedures. We report here a study aimed at developing improved systems for REE processing with the goal of efficient extraction of Th(IV) from acidic solution. A tripodal ligand, TRPN-CMPO-Ph, was prepared that utilizes carbamoylmethylphosphine oxide (CMPO) chelators tethered to a tris(3-aminopropyl)amine (TRPN) capping scaffold. The ligand and its metal complexes were characterized by using elemental analysis, NMR, Fourier transform infrared spectroscopy, mass spectrometry, and luminescence spectroscopy. Using a liquid–liquid metal extraction protocol, TRPN-CMPO-Ph selectively extracts Th(IV) at an efficiency of 79% from a mixture of Th(IV), UO_2_^2+^, and all rare-earth metal cations (except promethium) dissolved in nitric acid into an organic solvent. Th(IV) extraction selectivity is maintained upon extraction from a mixture that approximates a typical monazite leach solution containing several relevant lanthanide ions, including two ions at higher concentration relative to Th(IV). Comparative studies with a tris(2-aminoethyl)amine (TREN)-capped derivative are presented and support the need for a larger TRPN capping scaffold in achieving Th(IV) extraction selectivity.

## Abstract 2

A tripodal ligand based on the carbamoylmethylphosphine oxide (CMPO) chelator has been prepared and studied with regard to f-element coordination chemistry and metal extraction behavior. Utilizing a liquid−liquid extraction protocol, the ligand exhibits significant Th(IV) extraction selectivity when exposed to a mixture of Th(IV) and all rare-earth element cations, demonstrating its promise in the development of future metal separation applications.

Given their criticality to a variety of applications ranging from alternative energy to national security technologies, the rare-earth elements (REEs) and related metals are receiving more attention as supply chain concerns grow.^1−3^ Among the most pressing issues in REE acquisition is the metal separation problem stemming from the complex lanthanide/actinide (Ln/An) mixtures found within their minerals. For example, the monazite mineral, one of the most common raw sources of REEs, contains significant amounts of thorium(IV) [Th(IV)], a radioactive actinide metal ion, in most commercial ores,^4^ with some deposits possessing 30% Th(IV) content.^5^ Current procedures for extracting Th(IV) during initial REE mineral processing are inefficient, with poor metal extraction selectivity while generating large amounts of waste.^6,7^ There also exists a growing impetus to extract Th(IV) for its own applications including industrial catalysts^8,9^ and nuclear energy production.^10^ Improved methods for Th(IV) recovery would afford additional sources as interest grows in moving from uranium to Th-based reactors for clean energy applications.^11,12^

In this study, we developed a straightforward, selective Th(IV) extraction system that responds to the growing need for improved f-element separations. While promising Th(IV) extraction results have been documented using a variety of creative methods,^13−18^ these approaches remain limited by complex ligand synthetic procedures or extraction conditions requiring mixtures of additional chelators/ionic liquids, large extractant or nitric acid concentrations, or intricate protocols which may hinder practical application. Important to our approach is the utilization of the liquid–liquid extraction technique that is well-established for practical metal separation systems.^19,20^ When this process is applied in industry, the aqueous metal solution generated from the dissolution of raw materials is mixed with an organic solvent containing chelator molecules designed to bind the metal ions of interest and extract them into the organic solvent. Our approach described herein addresses the need for enhanced Th extraction from REE minerals without necessitating a major shift away from the well-established, scalable liquid–liquid method as we instead focus on the fundamental coordination chemistry contained within the process.

When considering f-element separation systems, the carbamoylmethylphosphine oxide (CMPO; Figure 1)^21,22^ chelating group is often employed. Since the development of this chelator for nuclear waste remediation via the *tr*ans-*u*ranium *ex*traction (TRUEX) process, efforts have been made tethering multiple CMPO groups to various scaffolds^23−26^ and solid supports^27^ to enhance metal extraction. These systems have added to the body of work focused on multipodal ligands for f-element separations;^28−31^ some of these studies have indeed demonstrated substantial Th(IV) extraction via liquid–liquid methods using pillar[5]arene-based phosphine oxide^28^ and tripodal nitrilotriacetamide ligands.^29^ Recent work in our laboratory has been geared toward developing preorganized, tripodal CMPO-based ligands using a modular synthetic approach that facilitates structural variation to tune the metal extraction selectivity. Since our initial report of enhanced Tb(III) extraction by the tris(2-aminoethyl)amine (TREN)-capped TREN-CMPO-OEt ligand (**1**, Figure 1),^32^ additional ligand caps and CMPO substituents have been explored (e.g., **2** and **3**, Figure 1),^33,34^ revealing relationships between structural variation and metal extraction.

**Figure 1:**
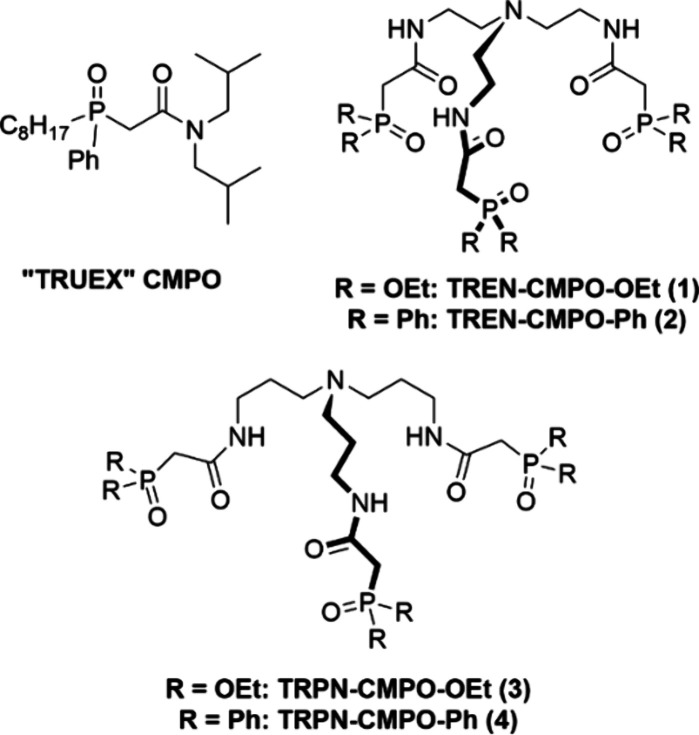
CMPO ligand used in the TRUEX process and tripodal CMPO ligands developed for enhanced Ln/An separations, including TRPN-capped ligand **4** prepared for this study.

This study focuses on a new extraction agent possessing the tris(3-aminopropyl)amine (TRPN) ligand scaffold, TRPN-CMPO-Ph (**4**, Figure 1), and describes its f-element extraction behavior. We pursued phenyl-substituted CMPO groups for ligand **4** to promote the solubility of the extracted metal complex in the organic phase. As noted in prior work with ethoxy-substituted ligands, moving from the TREN to TRPN capping scaffold in the case of ligand **3** led to a loss of Tb(III) selectivity and overall lower Ln extraction.^34^ While Ln(III) extraction was poor upon moving to the larger, more flexible TRPN cap, we were interested in assessing the effect of this structural change on Th(IV) extraction. Due to the relatively high charge density of this 4+ An ion versus 3+ Ln ions, the increase in the ligand flexibility afforded by the TRPN cap may allow the CMPO “arms” to fully wrap around the charge-dense Th(IV) and stabilize the metal–ligand complex upon extraction.

As shown in Scheme S1, ligand **4** was synthesized following established methods,^23,33−35^ condensing a *p*-nitrophenol-activated CMPO precursor with TRPN [see the Supporting Information (SI) for synthetic details]. To assess the f-element coordination chemistry in the solution and solid phases, 1:1 complexes of **4** with La(NO_3_)_3_ and Th(NO_3_)_4_ were prepared as solids and characterized by Fourier transform infrared, CHN elemental analysis, and NMR spectroscopy (see the SI). Analysis via IR reveals characteristic shifts of the ligand C=O and P=O stretches to lower wavenumbers, indicating coordination of the metal by both groups. Examination of the NMR spectra indicates that the resonance for the −CH_2_– group in the ^1^H NMR spectrum of the complexes shifts downfield relative to the ligand alone, as does the signal for the P=O group in the ^31^P NMR spectrum. Luminescence characterization was carried out for complexes of Eu(III) and Tb(III) in methanol to gain additional insight into the solution-state structure. As shown in Figure S1, ligand **4** acts as a sensitizer of Tb(III) and Eu(III) emission demonstrated by the characteristic narrow Ln-derived peaks. The observation of sensitized Ln(III) emission via the “antenna effect”^36^ supports metal complexation by **4** in solution.

Given the capability of TRPN-CMPO-Ph for effective metal complexation, extraction studies were next pursued. Benchmark liquid–liquid extractions conditions were employed including a 10:1 ligand-to-metal ratio, metal solutions prepared in 1 M HNO_3_(aq) (comparable with large-scale f-element separations), ligand solution in dichloromethane, and a phase mixing time of 20 h. Initially, extractions were performed utilizing individual metal solutions with analysis of the aqueous phase metal concentration by inductively coupled plasma optical emission spectroscopy to determine percent extraction values (Table 1). While ligand **4** exhibits low extraction efficiency for all Ln ions, a % *E* value for Th(IV) of 81.8 ± 0.1% was determined. It is worth noting that preorganization resulting from the TRPN-capped structure makes a significant difference in the Th(IV) extraction efficiency compared to a monopodal CMPO analogue, which extracts the An ion from 1 M HNO_3_ with a % *E* of ∼22% using a higher ligand-to-metal ratio of 70:1.^37^

**Table 1:** Percent Extraction Values for **4**^a^

metal	% *E*
La^3+^	3 ± 1
Ce^3+^	1.3 ± 0.4
Pr^3+^	4.0 ± 0.7
Nd^3+^	5.0 ± 0.6
Sm^3+^	3 ± 2
Eu^3+^	2.0 ± 0.4
Gd^3+^	1.7 ± 0.9
Tb^3+^	3 ± 2
Dy^3+^	2 ± 2
Ho^3+^	2 ± 1
Er^3+^	0.8 ± 0.5
Tm^3+^	3 ± 2
Yb^3+^	1.2 ± 0.4
Lu^3+^	1.9 ± 0.9
Th^4+^	81.8 ± 0.1

a Values were obtained by extracting metal from individually prepared 1 M HNO_3_ metal solutions into CH_2_Cl_2_; metal solutions were prepared at 1 × 10^–4^ M and ligand solutions at 1 × 10^–3^ M; 20 h mixing; room temperature.

Encouraged by the promising Th(IV) extraction results of TRPN-CMPO-Ph for individual metal solutions, we proceeded to assess the extraction selectivity by determining the ability of **4** to extract Th(IV) from an aqueous solution containing Th plus all rare-earth metals (including Sc and Y; excluding Pm) and uranyl. While select mixture extraction studies have been reported in the past,^16,25,38,39^ none to our knowledge include straightforward liquid–liquid extraction from such a complex mixture made up of 18 REE/An ions. Assessing the metal extraction selectivity from complicated mixtures is important for practical applications, especially for nuclear waste processing and raw source Th(IV) separations. Under the same conditions as those used for the individual metal extractions but now with extraction from a metal mixture solution, the extraction efficiency of Th(IV) remains high, at 79 ± 1%, while Ln(III) extraction is consistently low, with Ln % *E* values ranging from 3 to 6% (Figure 2). Additional extraction studies under similar conditions but with extraction from 3 M HNO_3_(aq) were also conducted for the metal mixture to assess the effect of increasing the acid strength. While the overall metal extraction increases as expected, with nearly quantitative extraction now seen for Th(IV), the difference in % *E* for Th(IV) versus Ln(III) was comparable with or larger than that of the 1 M results with % *E* values for Th(IV), Ce(III), Gd(III), and Yb(III) of 97.2 ± 0.2, 31 ± 2, 16 ± 1, and 12 ± 1%, respectively.

**Figure 2:**
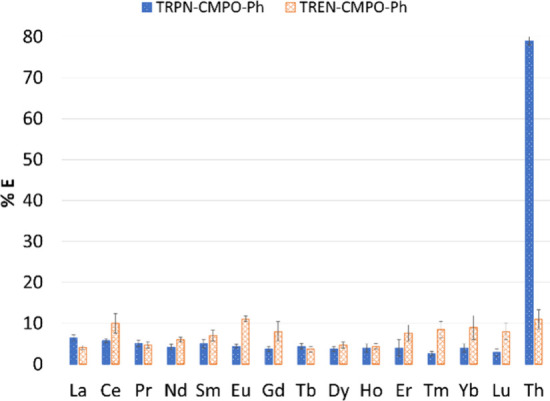
Percent extraction values for **4** (blue dotted) and **2** (orange crosshatched), extracting from a metal mixture solution containing Th(IV) all REE(III) ions (except Pm) and uranyl in 1 M HNO_3_ (% *E* values with standard deviations given in Table S1). The ligand solution was prepared at 1 × 10^–3^ M in CH_2_Cl_2_ and metal ion at 1 × 10^–4^ M; 20 h mixing; room temperature.

The significant difference in the extraction efficiency of Th(IV) versus all Ln ions represents unique selectivity, rarely seen under standard liquid–liquid extraction conditions, and can be attributed to the combination of the tripodal ligand design with the CMPO chelators tethered specifically to the TRPN cap. As seen in Figure 2, this capping scaffold effect is supported by a Th(IV) % *E* value of 11 ± 2% determined for the TREN-capped, phenyl-substituted ligand **2** under the same mixture extraction conditions (Ln extraction is also low for **2**, as evidenced by % *E* values for all Ln ions as shown). The relatively low extraction efficiency for TREN-capped **2** toward Th(IV) illustrates the importance of the TRPN capping scaffold in generating a cavity that preferentially binds and extracts Th(IV) compared to all Ln ions. Given the larger, more flexible TRPN cap relative to TREN, the resulting ligand structure appears to be better suited for encapsulating the charge-dense tetravalent ion and facilitating its extraction. This result is also consistent with a previously reported increase in the extraction of Pu(IV), another tetravalent actinide ion, by diglycolamide-based ligands upon moving from a TREN to more flexible TRPN capping scaffold.^40^

Finally, given the strong preference for Th(IV) extraction from a relatively complex Ln/An mixture, we assessed the extraction selectivity of **4** toward Th(IV) from a mixture closely approximating a standard REE mineral. The monazite mineral was chosen for this study due to its importance as a source of REEs and the typically high amounts of Th(IV) contained within. An acidic, mixed-metal solution was prepared containing Th(IV) and the four Ln ions present in the highest concentration within a typical monazite leach solution produced during mineral processing.^41^ Metal-ion concentrations ranging from 8.8 × 10^–7^ to 3.2 × 10^–5^ M, with relative concentrations matching those found within the raw source, were used along with the same ligand concentration as that in previous studies. As shown in Figure 3, with a smaller number of metal ions to compete with, Th(IV) is extracted at an efficiency of 77%, while the other metal ions are extracted at no more than 11% efficiency. It is noteworthy that pronounced Th(IV) selectivity is maintained despite its presence in the mixture at lower concentration relative to two other Ln ions present.

**Figure 3:**
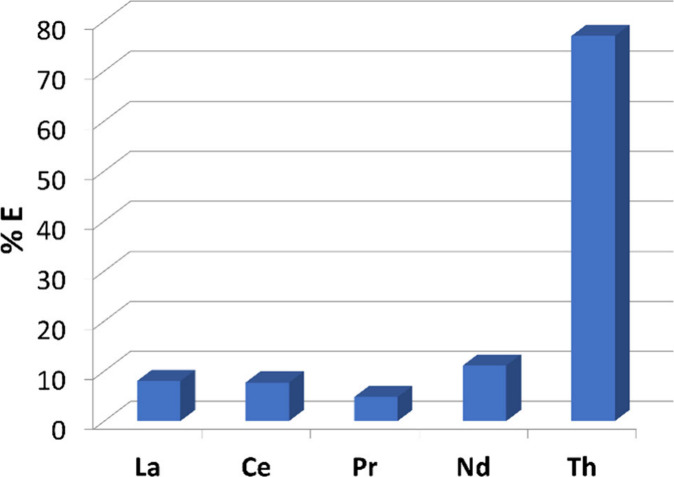
Percent extraction values for **4** extracting metal ions from a nitric acid solution of Th(IV) and select Ln(III) ions found in monazite (20 h mixing; room temperature). A ligand concentration of 1 × 10^–3^ M was used with concentrations of each metal chosen to approximate a typical mineral leach solution ([La(III)] = 3.0 × 10^–5^ M, [Ce] = 3.2 × 10^–5^ M, [Pr] = 8.8 × 10^–7^ M, [Nd] = 2.7 × 10^–6^ M, and [Th] = 2.0 × 10^–5^ M). Percent extraction values with standard deviations are given in Table S2.

In conclusion, a new tripodal CMPO-based ligand has been developed and assessed with regard to f-element coordination and separations. The TRPN-capped ligand **4** exhibits selective Th(IV) extraction from a mixture containing all REEs (except Pm) as well as uranyl, representing an extraction selectivity that is unique for systems utilizing the traditional liquid–liquid protocol. This selectivity is maintained upon the extraction of Th(IV) from a mixture approximating a known mineral composition. The direct comparison of ligands **2** and **4** underscores the importance of the TRPN capping scaffold for Th-selective extraction within this ligand series. Using the industry standard liquid–liquid extraction method along with a ligand that can be synthesized via a straightforward procedure renders this approach attractive for next-generation thorium extraction systems. Current efforts are underway to optimize the extraction protocol and gain further insight into this unique f-element extraction behavior.

## Supporting Information Available

Ligand and metal complex synthetic experimental details and related characterization data, luminescence and extraction experimental methods, and extraction efficiency results with associated error values for mixed metal extractions (PDF)
