# Supplementary material for: Selective Liquid–Liquid Extraction of Thorium(IV) from Rare-Earth Element Mixtures
Source: Inorg Chem. 2024 Jul 17;63(30):13815–9. doi: 10.1021/acs.inorgchem.4c01240 (PMC11289752; doi:10.1021/acs.inorgchem.4c01240)
Supplement: Supplementary file 1 — ic4c01240_si_001.pdf [file ic4c01240_si_001.pdf]

Selective Liquid-Liquid Extraction of Thorium(IV) from Rare Earth Element Mixtures

Wyatt B. Larrinaga[†], Bailey J. Lake[†], Victoria D. Pacanowski[†], Michael G. Patterson[†], Michael Hudson,[‡] Faith M. Carlson,[‡] Gabriel Heselschwerdt,[‡] Steven Balboa,[‡] Shannon M. Biros[‡], Eric J. Werner[†]*

[†] Department of Chemistry and Biochemistry, The University of Tampa, 401 W. Kennedy Blvd., Tampa, FL 33606; [‡] Department of Chemistry, Grand Valley State University, 1 Campus Dr., Allendale, MI 49401

Supporting Information

Experimental

General considerations

All chemicals were purchased from Sigma-Aldrich, Fisher Scientific, Strem Chemical, or Acros Chemicals and used without further purification. ^{1}H, ^{13}C and ^{31}P NMR spectral data were recorded on a JEOL ECZS 400 FTNMR spectrophotometer. For ^{1}H and ^{13}C NMR spectra, chemical shifts are expressed as parts per million (δ) relative to $SiMe_4$ (TMS, δ = 0), and referenced internally with respect to the protio solvent impurity. For ^{31}P NMR spectra, chemical shifts are expressed as parts per million (δ) relative to H_3PO_4 (δ = 0). Both ^{13}C and ^{31}P NMR spectra were obtained as proton-decoupled data. IR spectra were acquired neat on a Jasco 4100 FT-IR or a Thermo Nicolet Avatar 360 FT-IR spectrometer fitted with the ATR sampling accessory. Elemental (CHN) analyses were performed by Atlantic Microlab Inc., Norcross, GA, and luminescence spectra and lifetimes were measured using a Hitachi F-7000 spectrofluorimeter. High resolution mass spectrometry data were acquired by the Lumigen Instrument Center at Wayne State University. Extracted metal solutions were analyzed with a PerkinElmer Optima 2100 DV ICP-OES spectrophotometer or an Agilent 5800 ICP-OES. Synthesis of the TREN-CMPO-Ph ligand (**2**) for extraction studies was carried out following a previously reported procedure.[1]

Synthesis

Scheme S1. Synthetic procedure for TRPN-CMPO-Ph (**4**).

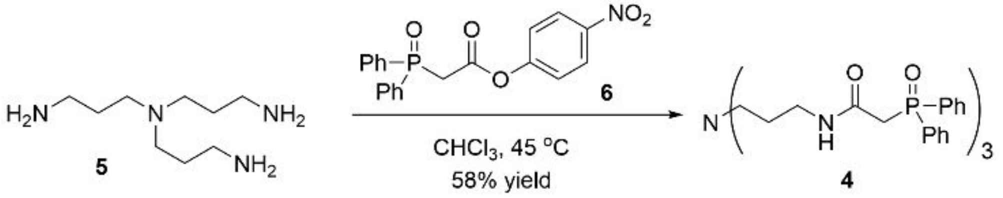

TRPN-CMPO-Ph (4). A 100 mL round bottom flask was charged with a magnetic stir bar, 1.73 g *p*-nitrophenol (diphenylphosphoryl) acetate[2] **6** (4.54 mmol) and 75 mL anhydrous chloroform (stabilized with amylenes). Tris(aminopropyl)amine **5** (0.214 g, 1.13 mmol) was added to a small vial, dissolved in a minimal amount of anhydrous chloroform, and added via syringe to the reaction mixture. The round bottom flask was equipped with a reflux condenser, rubber stopper and nitrogen balloon. The reaction mixture was heated to 45 ºC for three days, with stirring, and

allowed to cool to room temperature. Distilled water (50 mL) and 40% KOH (3-4 mL) was added, and the mixture was stirred for four hours at room temperature to hydrolyze any remaining *p*-nitrophenol ester. The mixture was transferred to a separatory funnel and the layers were separated. The organic layer was washed with water (3 x 20 mL) and brine (1 x 20 mL), dried over sodium sulfate and concentrated under reduced pressure. The resulting solid was triturated with diethyl ether (2 x 20 mL) and ethyl acetate (2 x 20 mL), then placed under high vacuum overnight to give the pure product as a tan powder (595 mg, 58 % yield). ^{1}H NMR ($CDCl_3$, 400 MHz): δ 7.80-7.65 (m, 4H), 7.55-7.35 (m, 6H), 3.34 (d, $J_{P\text{-}H}$ = 13.6 Hz, 2H), 3.14 (q, J = 6.0 Hz, 2H), 2.15 (broad, 2H), 1.41 (broad m, 2H); ^{13}C NMR ($CDCl_3$, 100 MHz): δ 164.8 (d, $J_{C\text{-}P}$ = 5 Hz), 132.4 (d, $J_{C\text{-}P}$ = 2 Hz), 131.4 (s), 131.0 (d, $J_{C\text{-}P}$ = 10 Hz), 128.9 (d, $J_{C\text{-}P}$ = 12 Hz), 50.8 (s), 39.1 (d, $J_{C\text{-}P}$ = 61 Hz), 37.8 (s), 26.4 (s); ^{31}P NMR ($CDCl_3$, 161 MHz): δ 30.1 ppm; ^{1}H NMR (3:1 CD_3CN/CD_3OD, 400 MHz): δ 7.40-7.73 (m, 10H), 3.01 (broad m, 2H), 2.16 (broad, 2H), 1.36 (broad m, 2H); ^{13}C NMR ($CDCl_3$, 100 MHz): δ 164.9 (d, $J_{C\text{-}P}$ = 8 Hz), 132.4 (s), 130.8 (d, $J_{C\text{-}P}$ = 16 Hz), 128.8 (d, $J_{C\text{-}P}$ = 19 Hz), 50.7 (s), 37.4 (s), 26.1 (s); ^{31}P NMR ($CDCl_3$, 161 MHz): δ 30.1 ppm; FT-IR (neat, cm^{-1}): *ν* 3269 (N-H), 1659 (C=O), 1175 (P=O); ESI-HRMS ($[M-H]^+$, *m/z*): calcd for $[C_{51}H_{57}N_4O_6P_3]\text{-}H^+$ 915.9683; found 915.3565.

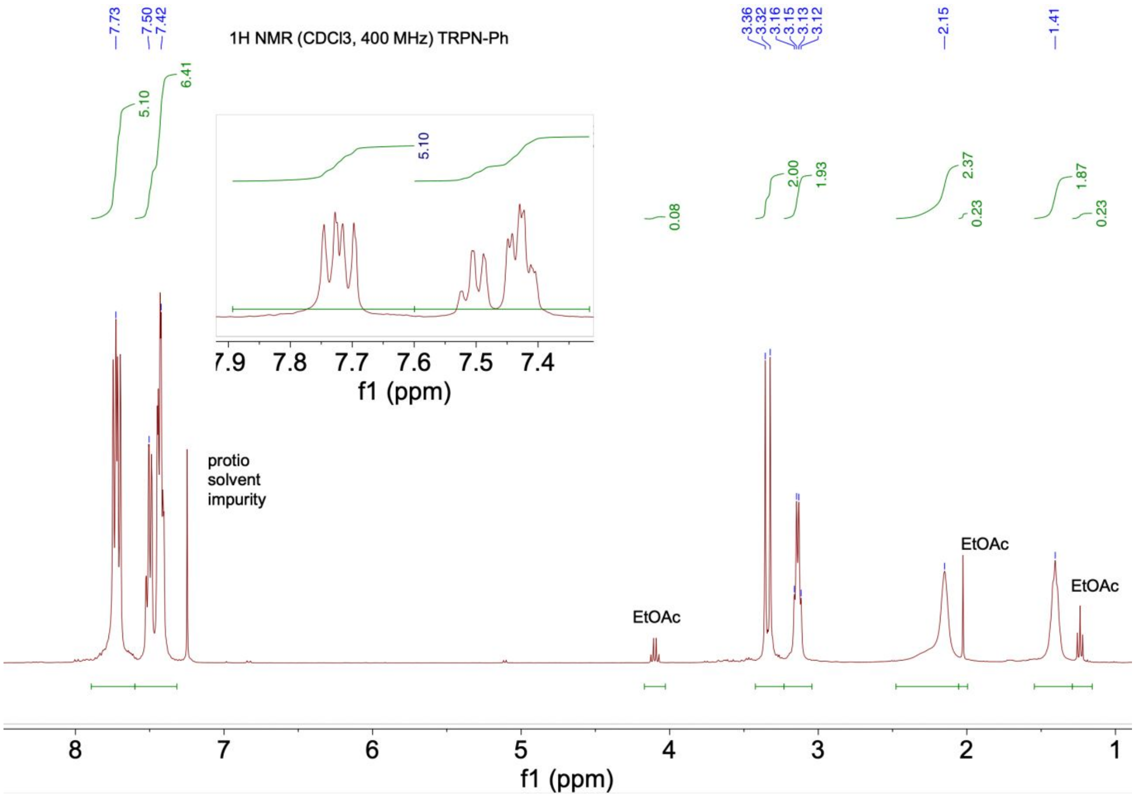

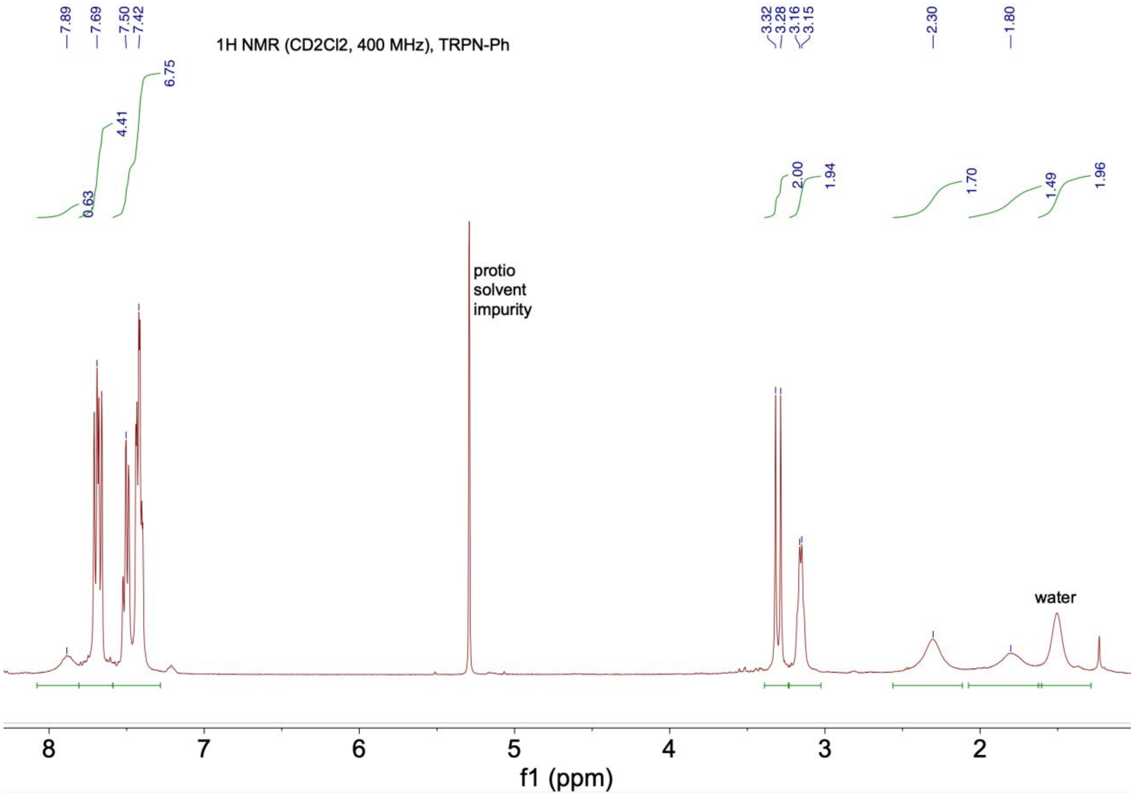
1H NMR (CD2Cl2, 400 MHz), TRPN-Ph
7.89
7.69
7.50
7.42
3.32
3.28
3.16
3.15
2.30
1.80
0.63
4.41
6.75
2.00
1.94
1.70
1.49
1.96
protio
solvent
impurity
water
8
7
6
5
4
3
2
f1 (ppm)

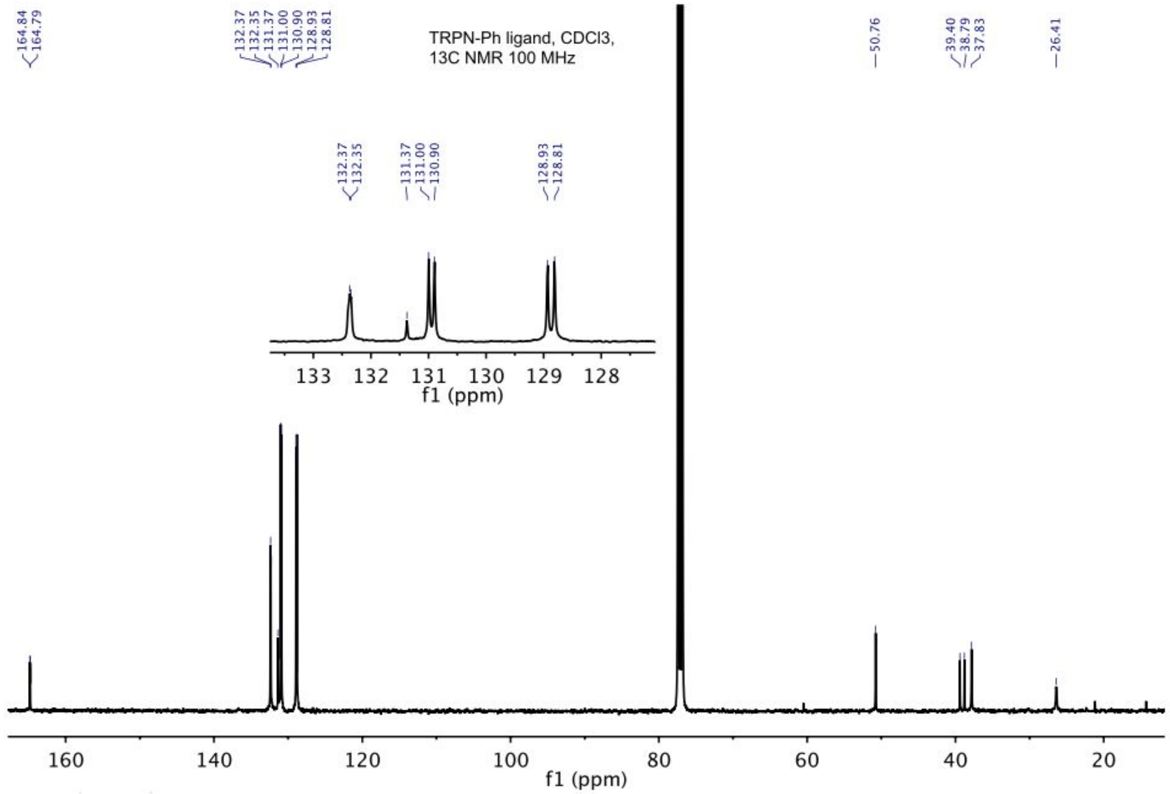
TRPN-Ph ligand, CDCl3,
13C NMR 100 MHz
164.84
164.79
132.37
132.35
131.37
131.00
130.90
128.93
128.81
50.76
39.40
38.79
37.83
26.41
133
132
131
130
129
128
f1 (ppm)
160
140
120
100
80
60
40
20
f1 (ppm)

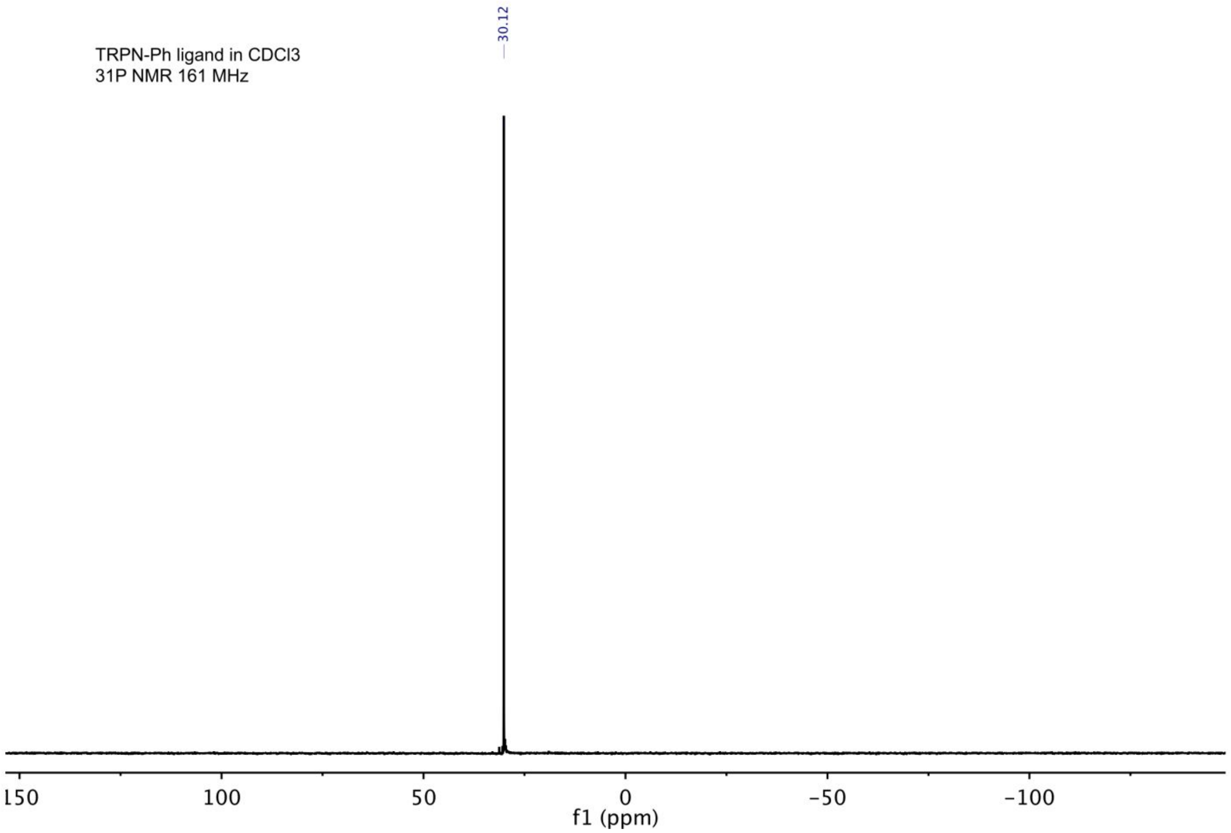

FT-IR TRPN-Ph (4, neat)

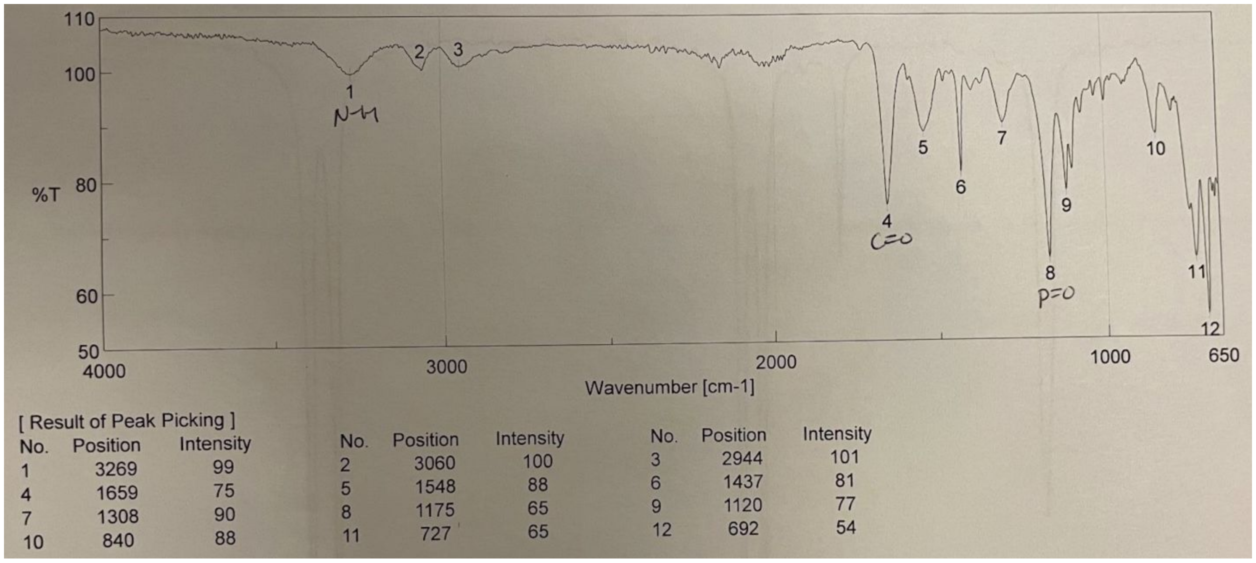

[Result of Peak Picking]

No.	Position	Intensity	No.	Position	Intensity	No.	Position	Intensity
1	3269	99	2	3060	100	3	2944	101
4	1659	75	5	1548	88	6	1437	81
7	1308	90	8	1175	65	9	1120	77
10	840	88	11	727	65	12	692	54

SB-TRPNPh #5-11 RT: 0.10-0.26 AV: 7 NL: 1.05E5
T: FTMS + c ESI Full ms [140.00-2000.00]

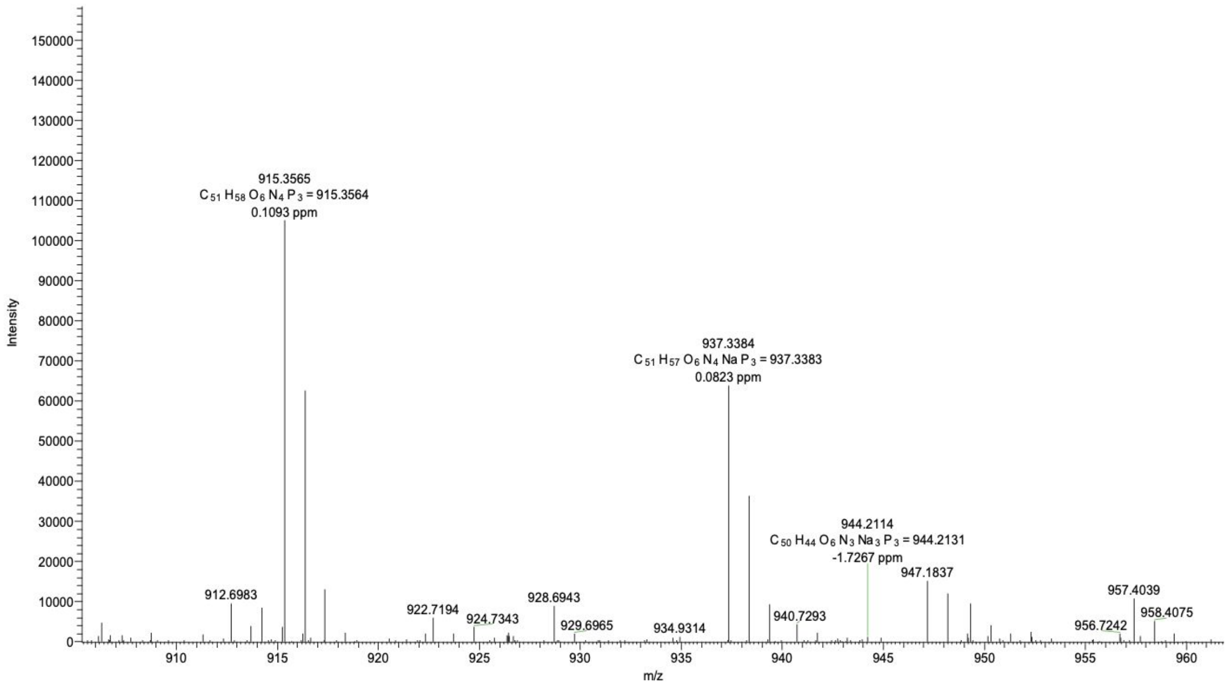

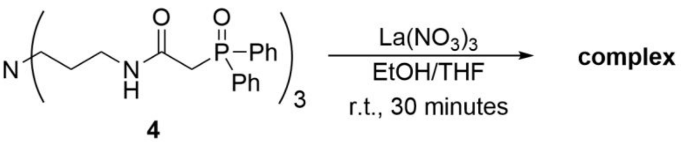

La(TRPN-Ph)(NO_3)$_3$. TRPN-CMPO-Ph (**4**) (200 mg, 0.219 mmol) was added to a 100 mL round bottom flask and dissolved in 20 mL of ethanol and 10 mL of THF. A magnetic stir bar was added, followed by lanthanum(III) nitrate hexahydrate (95 mg, 0.219 mmol) as a solid. The solution was stirred for 30 minutes at room temperature, at which point it was a milky white color. The solvents were removed under reduced pressure to give a solid residue, which was triturated with diethyl ether (2 x 10 mL) to give the product as a white powder (188 mg, 82 % yield). ^{1}H NMR (CD_3CN, 400 MHz): δ 7.87-7.71 (m, 4H), 7.58 (m, 2H), 7.47 (m, 4H), 3.82 (d, $J_{P\text{-}H}$ = 16 Hz, 2H), 3.03 (m, 2H), 2.12 (m, 2H), 1.22 (m, 2H); ^{31}P NMR (CD_3CN, 161 MHz): δ 35.3 ppm; FT-IR (neat, cm^{-1}): *v* 1625 (C=O), 1160 (P=O); Anal. Calcd. for [La($C_{51}H_{57}N_4O_6P_3$)(NO_3)$_3$(H_2O)$_5$] (found): C 46.06 (45.79), H 5.08 (4.46), N 7.37 (7.70).

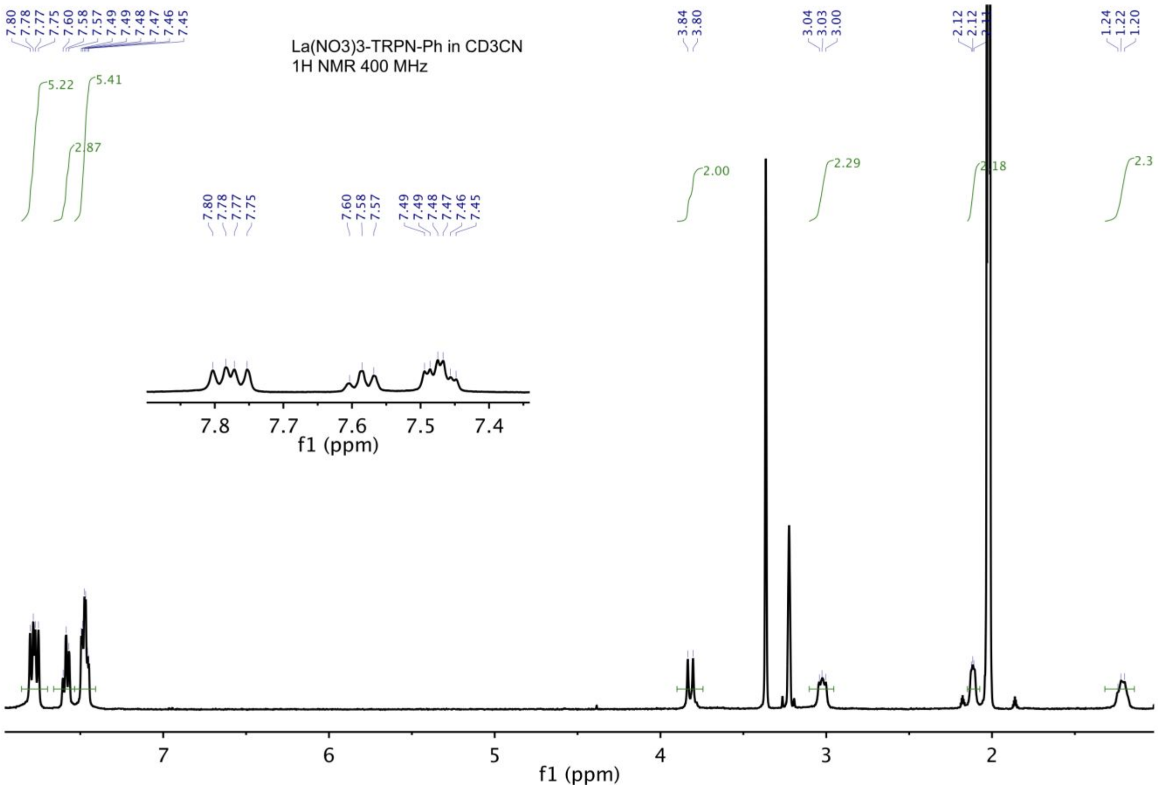

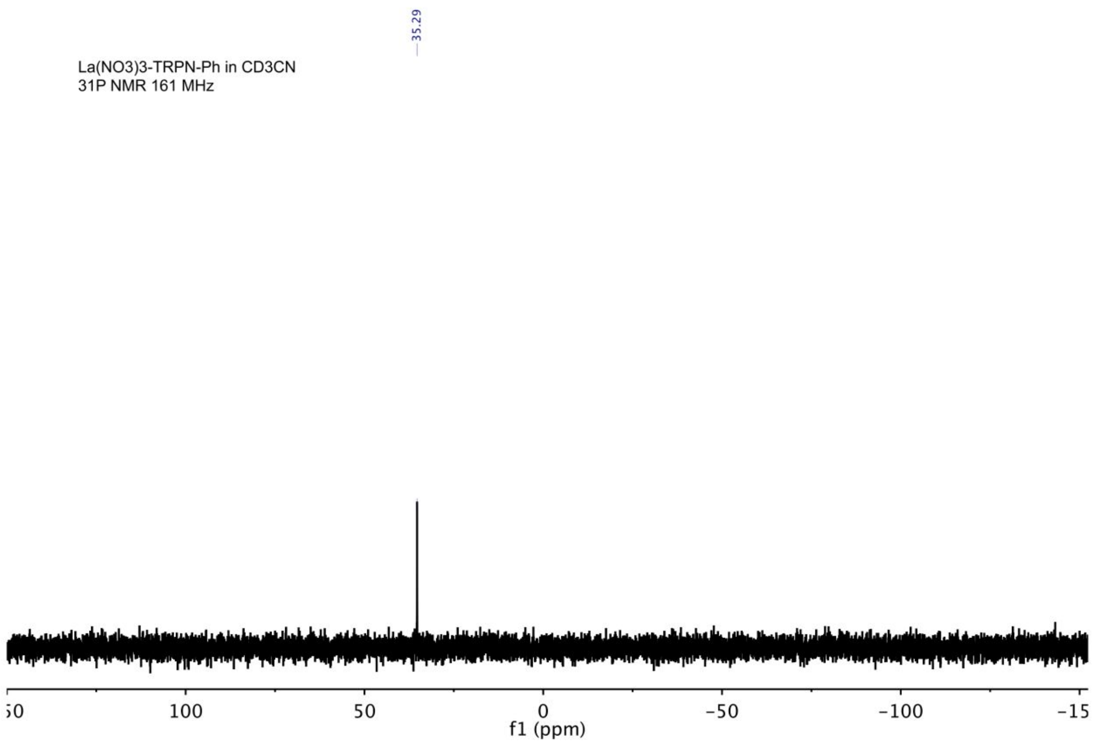

FT-IR La-TRPN-Ph (neat)

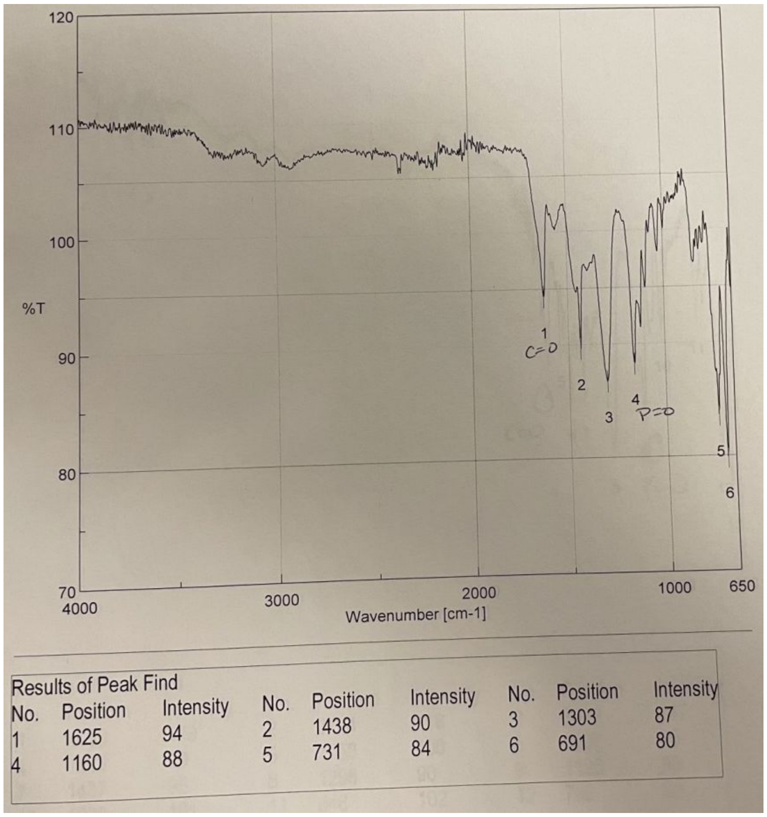

Results of Peak Find

No.	Position	Intensity	No.	Position	Intensity	No.	Position	Intensity
1	1625	94	2	1438	90	3	1303	87
4	1160	88	5	731	84	6	691	80

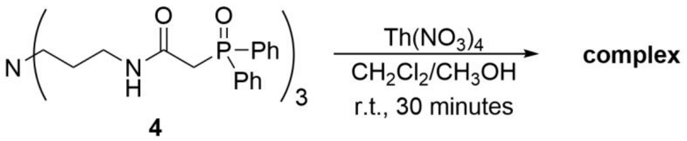

Th(TRPN-Ph)(NO_3)$_4$. TRPN-CMPO-Ph (**4**) (50 mg, 0.055 mmol) was added to a 50 mL round bottom flask containing a magnetic stir bar and dissolved in 10 mL of dichloromethane. Thorium(IV) nitrate hexahydrate (32 mg, 0.055 mmol) was added as a solid, followed by 5 mL of methanol which led to complete dissolution of the solids. The solution was stirred for 30 minutes at room temperature. The solvents were removed under reduced pressure to give a light tan powder, which was used without further purification. ^{1}H NMR (3:1 CD_3CN/CD_3OD, 400 MHz): δ 7.65-7.40 (m, 10H), 2.91 (broad, 2H), 2.11 (broad, 2H), 1.18 (broad, 2H), the N-*H* and C*H*$_2$ atoms have exchanged with deuterium; ^{13}C NMR (3:1 CD_3CN/CD_3OD, 100 MHz): δ 167.4 (d, $J_{C\text{-}P}$ = 6 Hz), 134.3 (s), 130.7 (d, $J_{C\text{-}P}$ = 11 Hz), 129.5 (d, $J_{C\text{-}P}$ = 14 Hz), 51.6 (s), 38.5 (s), 27.2 (s), the signal for the -*C*D$_2$- carbon atom is either obscured by the solvent or broadened into the baseline due to splitting from the nearby P and D atoms; ^{31}P NMR (3:1 CD_3CN/CD_3OD, 161 MHz): δ 42.1 ppm; FT-IR (neat, cm^{-1}): *v* 1620 (C=O), 1124 (P=O); Anal. Calcd. for [Th($C_{51}H_{57}N_4O_6P_3$)(NO_3)$_4$($C_4H_{10}O$)$_{1.5}$] (found): C 45.45 (45.79), H 4.82 (4.46), N 7.44 (7.70).

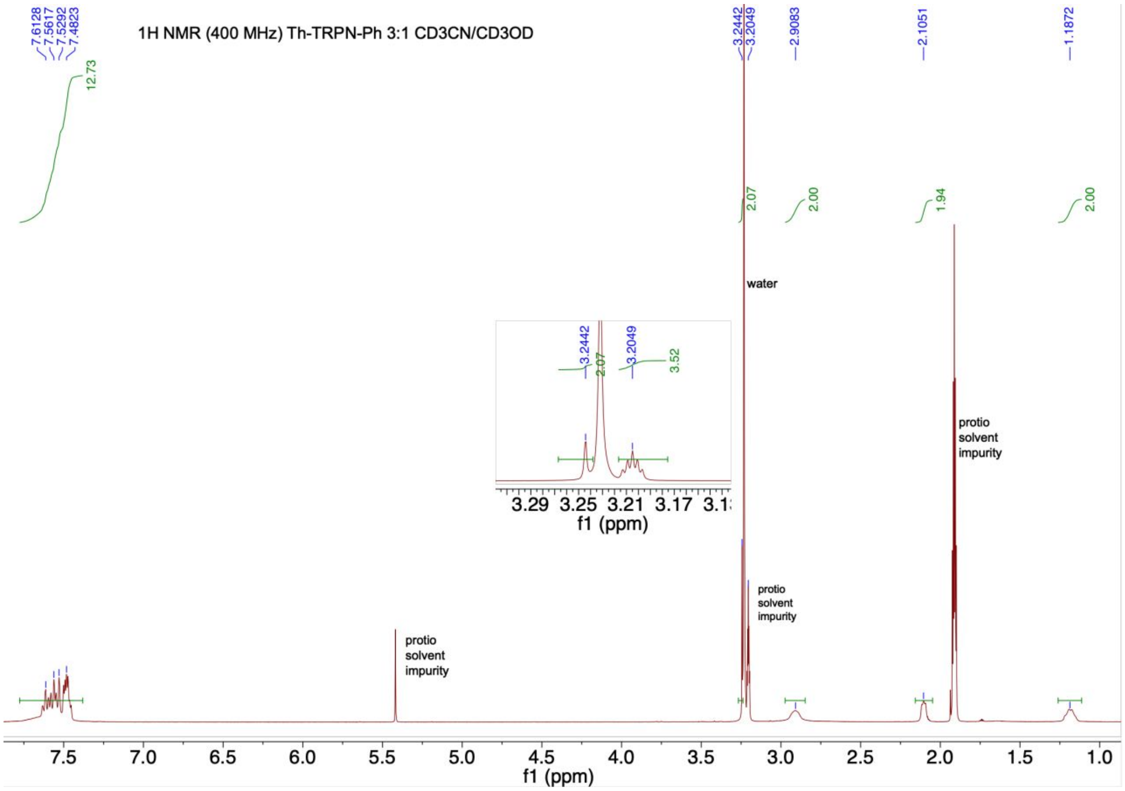

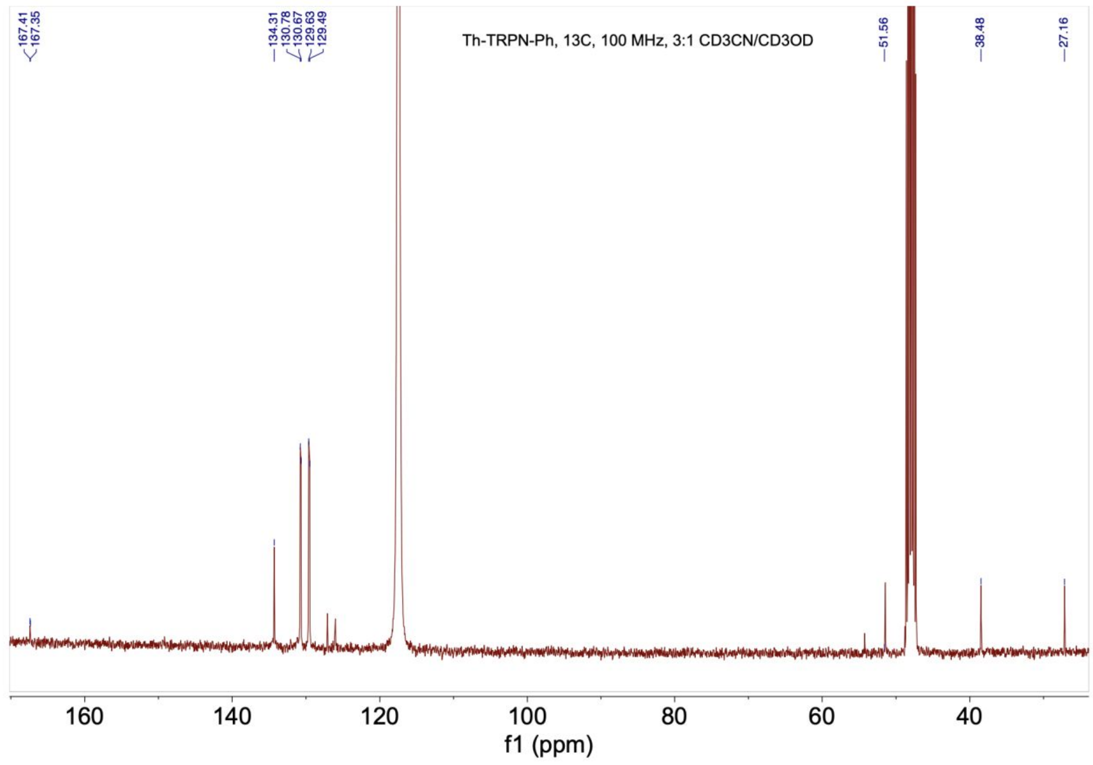
167.41
167.35
134.31
130.78
130.67
129.63
129.49
Th-TRPN-Ph, 13C, 100 MHz, 3:1 CD3CN/CD3OD
51.56
38.48
27.16
160
140
120
100
80
60
40
f1 (ppm)

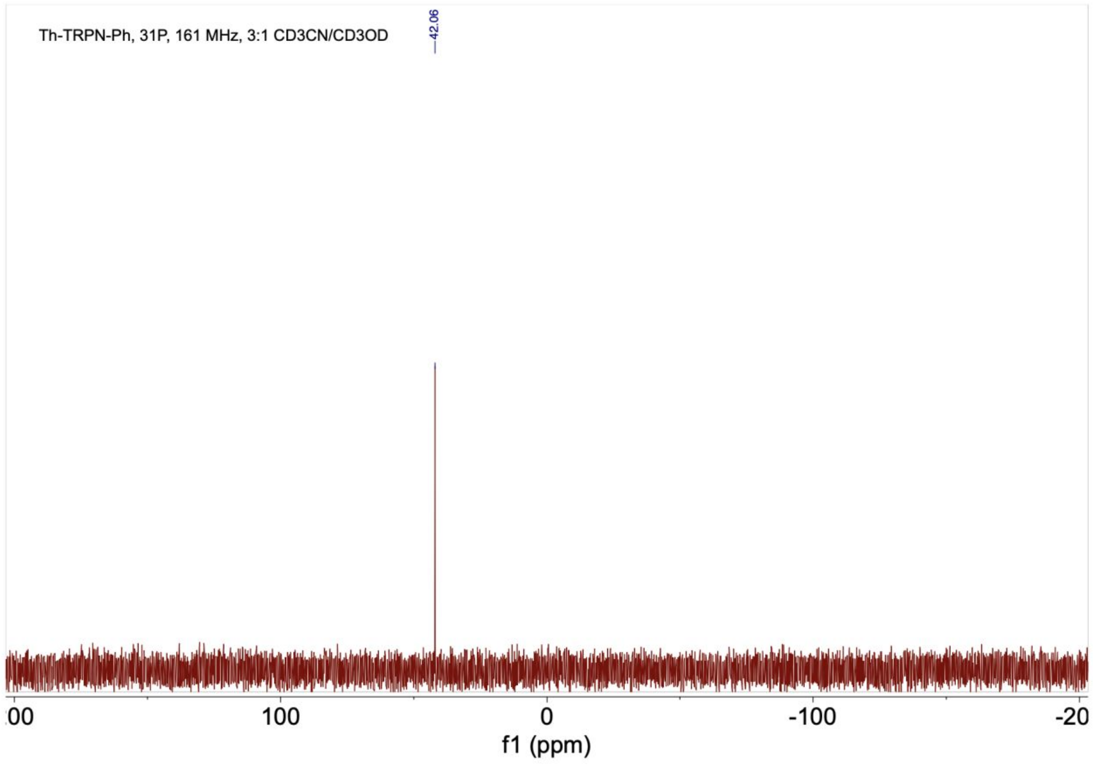
Th-TRPN-Ph, 31P, 161 MHz, 3:1 CD3CN/CD3OD
42.06
100
0
-100
f1 (ppm)

FT-IR Th-TRPN-Ph (neat)

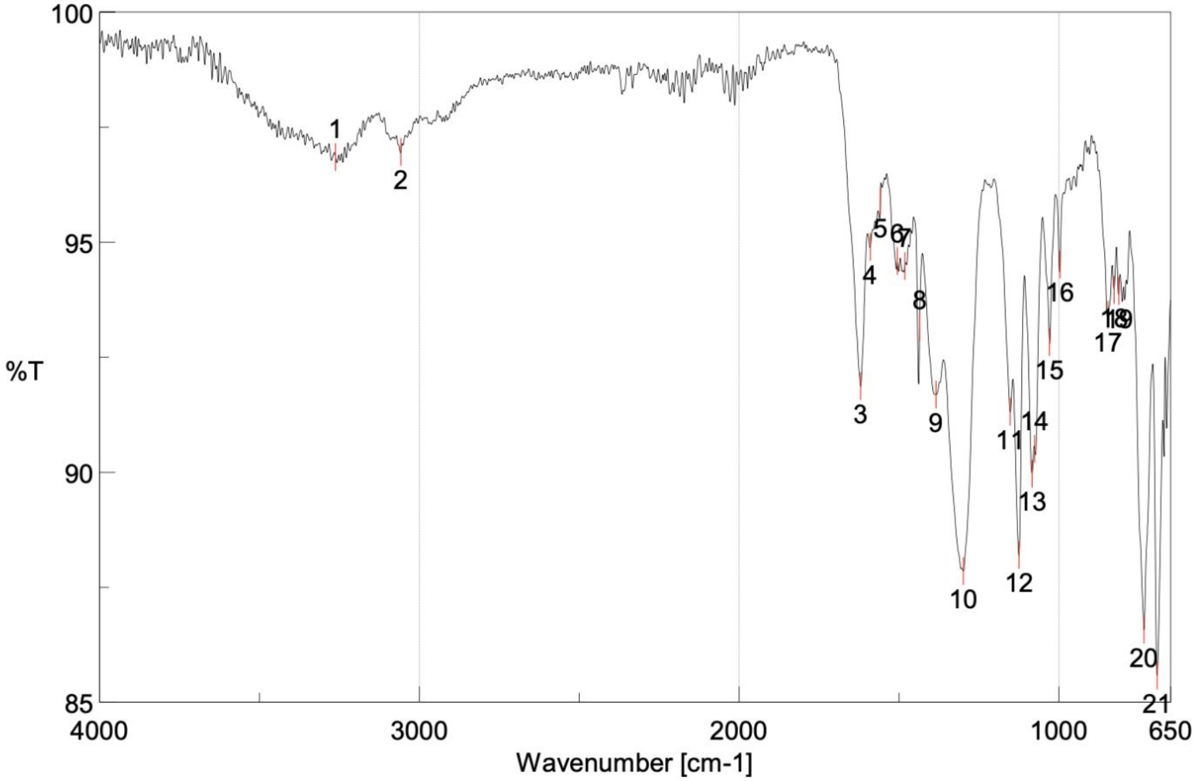

[Result of Peak Picking]

No.	Position	Intensity	No.	Position	Intensity
1	3262	97	2	3058	97
3	1620	92	4	1589	95
5	1558	96	6	1504	95
7	1481	94	8	1435	93
9	1384	92	10	1299	88
11	1152	91	12	1124	88
13	1083	90	14	1075	91
15	1029	93	16	996	95
17	847	93	18	827	94
19	812	94	20	734	87
21	692	86			

Luminescence studies

Eu(III) and Tb(III) complexes of TRPN-CMPO-Ph (**4**) were prepared in MeOH at 1.0×10^{-3} M with 5% ligand excess to ensure full metal ion complexation. Excitation and emission spectra for each Ln-**4** complex were recorded at room temperature with slit widths (ex/em) of 2.5 nm and a scan rate of 240 nm/min. The excitation spectra were recorded monitoring emission at 620 nm for Eu-**4** and 545 nm for Tb-**4**, and the emission spectra were recorded exciting the samples at 274 nm for Eu-**4** and 245 nm for Tb-**4**.

Extraction studies

Solutions of TREN-CMPO-Ph (**2**) and TRPN-CMPO-Ph (**4**) were prepared at 10^{-3} M in CH_2Cl_2. To achieve a 10:1 ligand to metal ratio for individual metal extraction experiments utilizing ligand **4**, metal solutions were prepared from La(III) and Ln(III) nitrate salts (penta/hexahydrate) for La, Ce, Pr, Nd, Sm, Eu, Gd, Tb, Dy, Ho, Er, Tm, Yb, and Lu at 10^{-4} M in 1 M HNO_3. An individual thorium(IV) solution was prepared by dilution of a 1000 ppm Th(IV) ICP-AES calibration standard solution (AccuStandard, Inc.) to 10^{-4} M in 1 M HNO_3. For the metal mixture extraction experiments with **2** and **4**, the mixed metal solution was prepared by diluting the ICP-AES rare earth standard mixture (AccuStandard, Inc.; 100 ppm Ln(III)) to 10^{-4} M in Ln(III) concentration in 1 or 3 M HNO_3; the same 10^{-3} M ligand concentration used for the individual metal extractions was used for the mixture experiments. For the targeted mixture experiments to approximate monazite leach solution, the mixed metal solution was prepared using Ln(III) nitrate salts of lanthanum, cerium, praseodymium, and neodymium with metal ion concentrations of 3.0 $\times 10^{-5}$, 3.2×10^{-5}, 8.8×10^{-7}, and 2.7×10^{-6} M, respectively, in 1 M HNO_3. Thorium(IV) from a 1000 ppm Th(IV) ICP calibration standard solution (AccuStandard, Inc.) was added to the mixed metal solution at a concentration of 2.0×10^{-5} M in 1 M HNO_3. The same 10^{-3} M ligand concentration as used for all other extraction studies was again used for the targeted mixture experiment.

All extractions were performed in triplicate for each individual metal and for the Th/Ln mixture experiments. For each experiment, a 20 mL scintillation vial was charged with equal volumes (2 or 4 mL) of each ligand and Th/Ln solution with all volumes measured using calibrated micropipettes. The combined solutions were stirred vigorously for 20 h at room

temperature, then allowed to separate completely. Post-extraction metal concentrations in the nitric acid phase were quantified by ICP-AES for determination of percent extraction values. From the extraction experiments prepared as outlined above using 4 mL each of aqueous and organic phase, 2.50 mL aliquots of extracted aqueous metal solutions were diluted to 25 mL in 3% HNO_3 and analyzed by ICP-AES (for 2mL aqueous/organic phase volumes, 1.00 mL aliquots were diluted to 10 mL). Pre-extraction reference samples were also prepared by diluting 2.50 mL aliquots of the metal solutions and diluting with 3% HNO_3 to 25 mL. Calibration samples of the Th/Ln metals were diluted to 1, 4, and 8 ppm from the rare earth standard solution. Wavelengths for ICP emission analysis of Th/Ln content were chosen to minimize interference due to other metal emissions as determined from literature references.[3,4] Concentrations of each metal ion were determined using the best-fit regression line produced from the calibration sample analysis. Percent extraction values were calculated using the following equation:

$$\%E = \frac{[Ln_0] - [Ln_{ex}]}{[Ln_0]} \times 100\ \%$$

where Ln_0 and Ln_{ex} represent pre-extraction and extracted Ln metal solution concentrations, respectively.

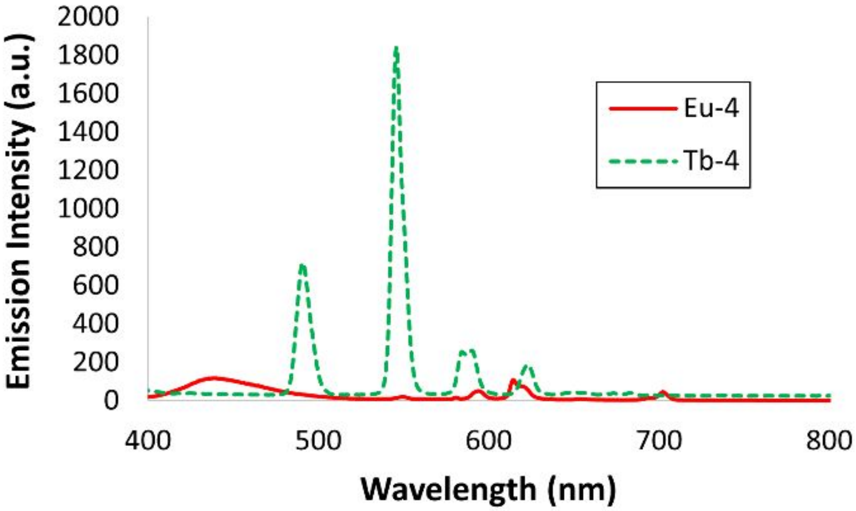

Figure S1. Luminescence emission spectra for the Eu(III) and Tb(III) complexes of **4** at 10^{-3} M in methanol; room temperature; excitation at 274 and 245 nm for the Eu and Tb complexes, respectively; slit widths of 2.5 nm.

Table S1. Percent extraction values (%*E*) for TREN-CMPO-Ph (**2**) and TRPN-CMPO-Ph (**4**). Values reported here represent averages from three independent extracton trials utilizing each ligand extracting each metal from a mixture of all rare earth metal ions plus Th(IV) and uranyl.[a]

Metal	%*E* via TREN-CMPO-Ph (**2**)	%*E* via TRPN-CMPO-Ph (**4**)
La^{3+}	4.1 ± 0.5	6.4 ± 0.8
Ce^{3+}	10 ± 2	5.8 ± 0.5
Pr^{3+}	4.7 ± 0.7	5.1 ± 0.8
Nd^{3+}	6.0 ± 0.6	4.2 ± 0.7
Sm^{3+}	7 ± 1	5 ± 1
Eu^{3+}	11.1 ± 0.7	4.4 ± 0.5
Gd^{3+}	8 ± 2	3.8 ± 0.6
Tb^{3+}	3.7 ± 0.7	4.4 ± 0.7
Dy^{3+}	4.7 ± 0.7	3.8 ± 0.6
Ho^{3+}	4.4 ± 0.7	4 ± 1
Er^{3+}	8 ± 2	4 ± 2
Tm^{3+}	9 ± 2	2.6 ± 0.6
Yb^{3+}	9 ± 3	4 ± 1
Lu^{3+}	8 ± 2	2.9 ± 0.9
Th^{4+}	11 ± 2	79 ± 1

[a] All extractions were performed at a 10:1 ligand to metal ratio with each ligand dissolved in CH_2Cl_2 (10^{-3} M) mixed with equal volumes of the mixed metal solution containing each individual metal ion at 10^{-4} M in 1 M HNO_3, stirring for 20 h at room temperature.

Table S2. Percent extraction values (%*E*) for TRPN-CMPO-Ph (**4**) extracting metals from targeted metal mixture including Th(IV), La(III), Ce(III), Pr(III), and Nd(III). Values reported here represent averages from three independent extracton trials.

Ln cation	%*E* via TRPN-CMPO-Ph (**4**)
La^{3+}	8 ± 1
Ce^{3+}	7.7 ± 0.9
Pr^{3+}	4.8 ± 0.5
Nd^{3+}	11.1 ± 0.9
Th^{4+}	77 ± 4

References

1. Patterson, M. G.; Mulville, A. K.; Connor, E. K.; Henry, A. T.; Hudson, M. L.; Tissue, K.; Biros, S. M.; Werner, E. J. *Dalton Trans.* **2018***, 47*, 14318–14326.
2. Arnaud-Neu, F.; Böhmer, V.; Dozol, J.; Grüttner, C.; Jakobi, R. A.; Kraft, D.; Mauprivez, O.; Rouquette, H.; Schwing-Weill, M.; Simon, N.; Vogt, W. *J. Chem. Soc., Perkin Trans. 2* **1996**, 1175–1182.
3. Mazzucotelli, A.; De Paz, F.; Magi, E.; Frache, R. *Anal. Sci.* **1992**, *8*, 189–194.
4. Liang, P.; Liu, Y.; Guo, L. *Spectrochim. Acta, Part B* **2005**, *60*, 125–129.
